# Associations of subjective and objective clinical outcomes of visual functions with quality of life in Chinese glaucoma patients: a cross-sectional study

**DOI:** 10.1186/s12886-019-1176-0

**Published:** 2019-07-31

**Authors:** Li Yang, Xuefeng Shi, Xin Tang

**Affiliations:** 1 Clinical College of Ophthalmology, Tianjin Medical University, Tianjin, 300020 China; 2 Tianjin Eye Hospital, Tianjin, 300020 China; 3 Tianjin Key Laboratory of Ophthalmology and Visual Science, Tianjin, 300020 China; 4 Tianjin Eye Institute, Tianjin, 300020 China

**Keywords:** Glaucoma, Quality of life, Pattern visual evoked potential, Visual acuity, Contrast sensitivity, Visual field, Rasch analysis

## Abstract

**Background:**

The cost of managing a chronic disease like glaucoma is quite large. A convenient and economical monitoring tool like self-reported measures of Quality of Life (QoL) could have the potential to significantly reduce the health economic burden of this disease. However, evidence about whether QoL can effectively reflect both subjective and objective clinical outcomes of visual functions in glaucoma patients is lacking. In this paper, we examined the relationships between both subjective and objective visual functions and QoL in glaucoma patients.

**Methods:**

This cross-sectional study enrolled 107 patients with a broad range of glaucomatous visual function loss. Subjective visual function loss was assessed using tests of visual acuity (VA), contrast sensitivity (CS) and visual field (VF). Evaluation of objective visual function was performed using pattern visual evoked potentials (PVEP). National Eye Institute Visual Function Questionnaire 25 (NEI VFQ-25) and Glaucoma Quality of Life-15 (GQL-15) were used to measure QoL. Uni- and multivariable linear regression analyses were performed to investigate the associations between all the clinical variables with Rasch-calibrated QoL scores.

**Results:**

Univariate analysis revealed that worse Rasch-calibrated NEI VFQ and GQL scores were associated with poorer VA and CS in both the better eye (BE) and the worse eye (WE), and with worse VF mean deviation, VF pattern standard deviation and PVEP latency and amplitude in 15 min check size in the WE (*P* < 0.05). Multivariable linear regression analysis revealed that, after adjusting for age, gender, duration of glaucoma, glaucoma severity and glaucoma type, Rasch-calibrated NEI VFQ-25 person measure scores were significantly associated with PVEP latency in 15 min check size [*β* = − 0.347, 95% *CI*: (− 0.618, − 0.118), *P* = 0.001] in the WE; Rasch-calibrated GQL-15 person measure scores were significantly associated with PVEP amplitude in 15 min check size [*β* = − 0.338, 95% *CI*: (− 0.588, − 0.108), *P* < 0.001] in the WE.

**Conclusions:**

Self-reported measures of QoL could be supplemental tools for assessing both subjective and objective visual functions in glaucoma patients.

**Electronic supplementary material:**

The online version of this article (10.1186/s12886-019-1176-0) contains supplementary material, which is available to authorized users.

## Background

Glaucoma is a leading cause of irreversible loss of vision worldwide, which currently affects over 61 million individuals in the world. The affected population is expected to increase to about 80 million by 2020, in which Asians will represent a large part of those [1]. Epidemiological investigation revealed that the suffering population of glaucoma in China is expected to increase to about 21 million by 2020 [2].

The impact of glaucoma on individuals’ visual functions can be assessed by subjective clinical examinations or objective clinical tests [3]. However, many patients present normal VA initially, although glaucoma is progressively and adversely affecting their QoL. Traditionally, glaucoma was thought to mainly affect peripheral visual function, especially in its early stage. Peripheral vision loss can be detected with VF test, but we need to have repeat examinations for confirming disease progression [4]. Furthermore, researchers found that some patients do not complain their vision loss as a black tunnel effect or as black patches, but complain about blurred patches or missing patches [5]. Indeed, CS impairment reported in glaucoma patients was even earlier than visual acuity impairment [6, 7]. Richman et al. reported that VA and CS are better predictors than VF in assessing QoL [8], but Ekici’s report placed more importance on CS and VF mean deviation than other clinical measures [9]. In one word, the tests for VA, CS and VF may have different superiority and insufficiency, respectively. On the other hand, they are all subjective tests, which need patients’ input and are easily influenced by patients’ comprehension, emotion and psychological state. Visual evoked potential, as an objective visual functional test, provides a unique tool to assess the visual functional impairment in glaucoma patients [10–12]. Specifically, PVEP has been shown increasing abnormalities as glaucomatous optic nerve damage progressed [13]. Therefore, multiple tests are necessary and beneficial to the diagnosis, treatment and monitoring of glaucoma.

Given that the cost of managing a chronic disease like glaucoma is quite large [14], worsening disease severity is accompanied by greater consumption of social resources [15], leading to a heavier burden. A convenient and economical monitoring tool could solve this problem to a large extent. Questionnaire-based self-reported assessments such as the 25-item National Eye Institute Visual Function Questionnaire-25 (NEI VFQ-25) [8, 16–22] and the 15-item Glaucoma Quality of Life-15 (GQL-15) [23, 24] have long been used to estimate the influence on glaucoma patients’ QoL, and showed relationships with subjective clinical outcomes, mainly of VF and / or VA [8, 16, 18–24]. On the other hand, although PVEP has been used as a unique objective tool to assess the visual functional impairment in glaucoma patients [11–13], no study has ever examined the association between the PVEP outcomes and patients’ QoL.

Therefore, in this study, we systematically explored the associations of both subjective and objective clinical outcomes of visual functions with QoL in a same group of Chinese glaucoma patients.

## Methods

### Design and setting

This was a cross-sectional study. The study was approved by the Ethics Committee of Tianjin Eye Hospital (Registration Number TJYYLL-2016-20). Written informed consent was obtained from all participants in accordance with the Declaration of Helsinki. All the participants diagnosed of primary open-angle glaucoma (POAG) or chronic angle-closure glaucoma (CACG) with a broad range of visual function loss were recruited at Tianjin Eye Hospital, a large university teaching hospital which serves patients from a large area. The diagnosis was in accordance with the Preferred Practice Pattern Guidelines by American Academy of Ophthalmology and the China Expert Consensus on the Diagnosis and Treatment of Primary Glaucoma (Version 2014) by Chinese Ophthalmological Society. Other inclusion criteria included age between 27 and 88 years and the ability to understand and speak Chinese.

Exclusion criteria included: 1) any eye disease that causes visual impairment other than glaucoma, 2) neurological or musculoskeletal diseases, such as dementia that would have effect on daily living activities and keep patient from providing reliable and valid data, 3) incisional eye surgery within the past 6 months, 4) laser therapy within the previous month, 5) refractive errors greater than 5 dioptres sphere or 2 dioptres cylinder, or visually significant cataracts (greater than Stage 2 LOCS III classification).

Demographic information was collected at the time of enrollment. Each patient underwent comprehensive clinical examinations including slit lamp biomicroscopy, intraocular pressure, fundus examination, VA, CS, VF, and PVEP.

### Assessment of QoL

QoL was assessed with two questionnaire instruments, NEI VFQ-25 questionnaire and (eTable 1 in Additional file 1) GQL-15 questionnaire. NEI VFQ-25 is most commonly used in ophthalmic clinic. It consists of 12 subscales including five non-visual domains (general health, mental health, dependency, social function, role difficulties) and seven visual domains (general vision, distance vision, peripheral vision, driving, near vision, colour vision, and ocular pains). We scored the responses of NEI VFQ-25 from 1 (worst QoL) to 5 (best QoL), with the score 0 coded as missing data. Some category responses were reversed for Rasch analysis so that the polarity would be the same for all included items. The GQL-15 covers four domains: central and near vision, peripheral vision, glare and dark adaptation, and outdoor mobility [24–26]. We scored the responses of GQL-15 from 1 (no difficulty at all, best QoL) to 5 (severe difficulty, worst QoL) [26].

We used Chinese versions of NEI VFQ-25 and GQL-15 questionnaires, which were translated from original English version, and which have been proven to have good reliability and validity for assessment of QoL in Chinese glaucoma patients [25–27].

### Rasch analysis of QoL

Rasch analysis was performed using Winsteps software (Version 3.72.3, J.M. Linacre, Chicago, IL, available at www.winsteps.com) to check the validation and the psychometric properties of these two questionnaires, and to calculate person measures of each participant. The unit of this measure is called a logit (log-odds), and it enables us to place participants according to their ability on the same linear interval scale.

Prior to Rasch analysis, floor effect or ceiling effect were tested for all the items of the NEI VFQ-25 and the GQL-15. Items with 80% or more of responses as ‘None’/‘Not at all’ or ‘Almost always’ / ‘Severely’/ ‘Extremely’, were eliminated. Item ‘*Color vision*’ from the NEI VFQ-25 was removed for a strong floor effect. We examined the ordering of thresholds to determine whether successive integer scores increased for the measured construct. All category thresholds were ordered properly. Afterwards, we calculated fit statistics of the data to determine fit to the Rasch model. Items with mean-square of infit or outfit values < 0.50 or > 1.50 were considered for removal. Ten items from NEI VFQ-25 and three items from GQL-15 were removed for not fit into the model. Unidimensionality was tested by principal component analysis (PCA) of the residuals. Data are considered unidimensional if the variance explained by the principal component exceeds 60%, at the meantime, the contrast to the principal component should be < 2.0 Eigenvalue units. The PCA of the residuals explained 53.8% of the raw variance in the GQL-15, and the unexplained variance by the first contrast of the residuals was 2.5 eigenvalues units in the NEI VFQ-25. This implies that these instruments were multidimensional. Thus, based on the result of the PCA and different types of visual skills, we formed four separate scales for NEI VFQ-25 (*‘General vision and social function’, ‘Far vision’, ‘Outdoor’, ‘Reading and working’*) (eTable 2 in Additional file 1) and four separate scales for GQL-15 (*‘Details identification’, ‘Walking’, ‘Adjusting to lights’, ‘Difficult task’*) (eTable 3 in Additional file 1), respectively. All the items in these separate scales showed satisfied fit to the model (eTables 2 and 3 in Additional file 1), and PCA of these scales revealed unidimensionality (eTables 4 and 5 in Additional file 1). Differential item functioning (DIF) was assessed for age (≤64 years, > 64 years), gender, duration of glaucoma (≤3 years, > 3 years) and glaucoma type. All the scales did not show notable DIF. The overall performance of the model was evaluated using person separation indices (e.g. reliability coefficient) and targeting (difference between mean person measure and mean item measure). A person separation index of 1.50 represents an acceptable level of separation. Person separation indices range from 1.52 to 2.21 for all the scales. Finally, person measure data were rescaled from the original logit scale to a user-friendly, but still linear, scale ranging from 0 to 100. A higher person measure score indicates a higher level of vision functioning in the Rasch-calibrated NEI VFQ and worse condition of visual impairment in the Rasch-calibrated GQL. A single average score of four person measure scores in each questionnaire was calculated to characterize the overall QoL.

### Measures of subjective clinical variables

Best corrected VA was monocularly measured using an Early Treatment Diabetic Retinopathy (ETDRS) chart. VA was scored with logarithm of the minimum angle of resolution. CS was tested with CSV-1000E (Vector Vision, Haag-Streit, Harlow, UK). There are 4 different spatial frequencies in the CS test: 3 cycles/degree (cdp), 6 cdp, 12 cdp and 18 cdp. The chart was back-illuminated and viewed from 3.5 m with mean luminance of 85 cd/m^2^ (low photopic condition). A single quantity, the area under the log CS function (AULCSF), was calculated to characterize the overall CS function. The AULCSF result was integrated between the fixed limits of log spatial frequencies of 0.48 (corresponding to 3 cpd) and 1.26 (corresponding to 18 cpd). The Humphrey 24–2 Swedish Interactive Threshold Algorithm (SITA) Standard perimeter (Carl Zeiss Meditec, Dublin, CA) was used to test VF. The main indices of the Humphrey perimetry are mean deviation (MD) and pattern standard deviation (PSD).

Definitions of the BE and the WE are described in eTable 6 in Additional file 1. The severity of glaucoma was classified on the basis of MD in the WE visual field as mild (− 2.00 to − 10.00 dB), moderate (− 10.01 to − 20.00 dB) and severe (< − 20.00 dB).

### Measures of pattern visual evoked potentials

PVEP recordings were performed using Roland-Consult RETIport system (Wiesbaden, Germany) on the basis of International Society for Clinical Electrophysiology of Vision (ISCEV) standard. Subjects were seated 1 m in front of a display (a 17 in. screen) in a dark and quite room. The screen edge subtended 15°of visual angle. Pattern-reversal stimuli were presented as full-field black and white checkerboard with large 1 degree (°) (i.e., 60 min of arc (min)) and small 0.25° (15 min) checks reversed at the rate of 2 reversals per second, no transient luminance or contrast change (contrast, 80%; mean luminance, 58 cd/m^2^). A small fixation target (a red cross), subtending a visual angle of approximately 0.5°, was placed at the center of the display, and was changed into a red point of the same size when participants declared that they could not clearly perceive the fixation target. The recording was conducted after correcting refractive error. Stimulation was monocularly given after masking the other eye. Gold cup skin electrodes were fixed in the following positions: the active electrode (Oz) was placed 3 cm above the middle point of occipital protuberance, the reference electrode (Fpz) was clipped onto the left ear-lap, and the neutral electrode (fpz) was attached to the forehead. All electrodes were set up to an impedance of less than 10 k-ohm. The analysis time was 350 ms (70 reversals). During recording, the patient’s fixation was strictly fixed on the fixation target. During a recording session, each eye was recorded at least 3 times, and we determine the most repetitive wave as the result wave. Each participant underwent at least 2 recording sessions at least 1 day apart, to determine test-retest variability. PVEP signals with a signal-to-noise ratio of > 2 was accepted. In this study, both the better eye and the worse eye were tested and the amplitudes and latencies of P100 were analyzed [28].

### Statistical analysis

Data were entered into Microsoft Excel and were later uploaded and analyzed using the Statistical Package for the Social Sciences (SPSS, version 17). Demographics, QoL scores, and clinical outcomes were summarized using means, standard deviations, medians, and ranges or as proportions, frequencies and percentages when appropriate. Student’s *t* test was used for comparison of means in normally distributed data of continuous variables. We used ANOVA to compare means of mild, moderate and severe glaucoma participants. Mann-Whitney’s *U* test was conducted to compare means in non-normally distributed data of continuous variables. Chi-square test was used to compare the frequency difference between groups. We performed univariate linear regression analysis to assess the possible correlations of clinical variables with QoL. Subsequently, stepwise multivariable linear regression was performed for all variables found to be significantly associated with QoL in the correlation analysis. *t* test based 95% confidence intervals for *β* regression coefficients unadjusted and adjusted for age, gender, duration of glaucoma, glaucoma severity, and glaucoma type were calculated. A two-tailed *P* value of < 0.05 was considered to be statistically significant.

## Results

### Demographics and clinical outcomes of the participants

A total of 214 eyes of 107 glaucoma patients were examined in this study. The mean (SD) age was 61.94 (±10.95) years, the median age was 64 years, the mean (SD) duration of glaucoma was 3.56 (±2.35) years, 42.99% of the subjects (46/107) were male. The sociodemographic characteristics and the results of subjective and objective clinical outcomes of the 107 participants are summarized in Table 1. The Rasch-calibrated NEI VFQ and the Rasch-calibrated GQL displayed good ordered thresholds, infit and outfit, with no multidimensionality and DIF. In the meantime, the Rasch-calibrated NEI VFQ scores decreased (i.e., activity ability worsened) with increasing glaucoma severity among groups (*P* = 0.001, eTable 7 in Additional file 1); the Rasch-calibrated GQL scores increased (i.e., activity limitation increased) with increasing glaucoma severity among groups (*P* = 0.001, eTable 7 in Additional file 1), indicating good criterion validity of these two QoL instruments. The sociodemographic characteristics and the results of Rasch-calibrated QoL scores of all the participants and the subgroups of different severity are summarized in eTable 7 in Additional file 1.

**Table 1:** Sociodemographic and clinical characteristics of the participants

Variables	Participants			
Number of participants*	107		–	
Sex				
Male	46		42.99%	
Female	61		57.01%	
Age (years)	61.94 (10.95)		27 to 81	
Duration of glaucoma (years)	3.56 (2.35)		2 to 21	
Type of glaucoma				
CACG	74		69.16%	
POAG	33		30.84%	
	Better eye		Worse eye	
Visual acuity (logMAR)	0.17 (0.21)	−0.14 to 0.96	0.37 (0.3)	− 0.1 to 1.3
Contrast sensitivity	0.98 (0.28)	0.33 to 1.56	0.78 (0.34)	0.33 to 1.48
Visual field				
MD (dB)	−4.95 (5.3)	−28.99 to 1.02	−13.42 (9.4)	−32.28 to − 2.01
PSD (dB)	3.21 (2.6)	0.96 to 12.93	6.11 (3.84)	1.27 to 14.81
PVEP (*n* = 60)				
Latency in 1 deg. size (ms)	108.72 (10.81)	90.4 to150	115.6 (16.02)	90.4 to 150
Amplitude in 1 deg. size (μV)	11.08 (5.04)	0 to 31.9	8.16 (5.15)	0 to 30
Latency in 15 min size (ms)	123.07 (13.69)	99.2 to 150	130.52 (15.66)	100.4 to 150
Amplitude in 15 min size (μV)	13.94 (8.79)	0 to 34.7	8.17 (6.82)	0 to 32.6

Data are presented as mean (standard deviation) unless otherwise indicated; ^*^: *N* = 107 for all variables except for PVEP (N = 60); *CACG* chronic angle-closure glaucoma, *POAG* primary open-angle glaucoma, *logMAR* logarithm of the minimum angle of resolution, *MD* mean deviation, *PSD* pattern standard deviation, *PVEP* pattern visual evoked potentials

### Associations between subjective and objective visual functions and QoL

**Fig. 1:**
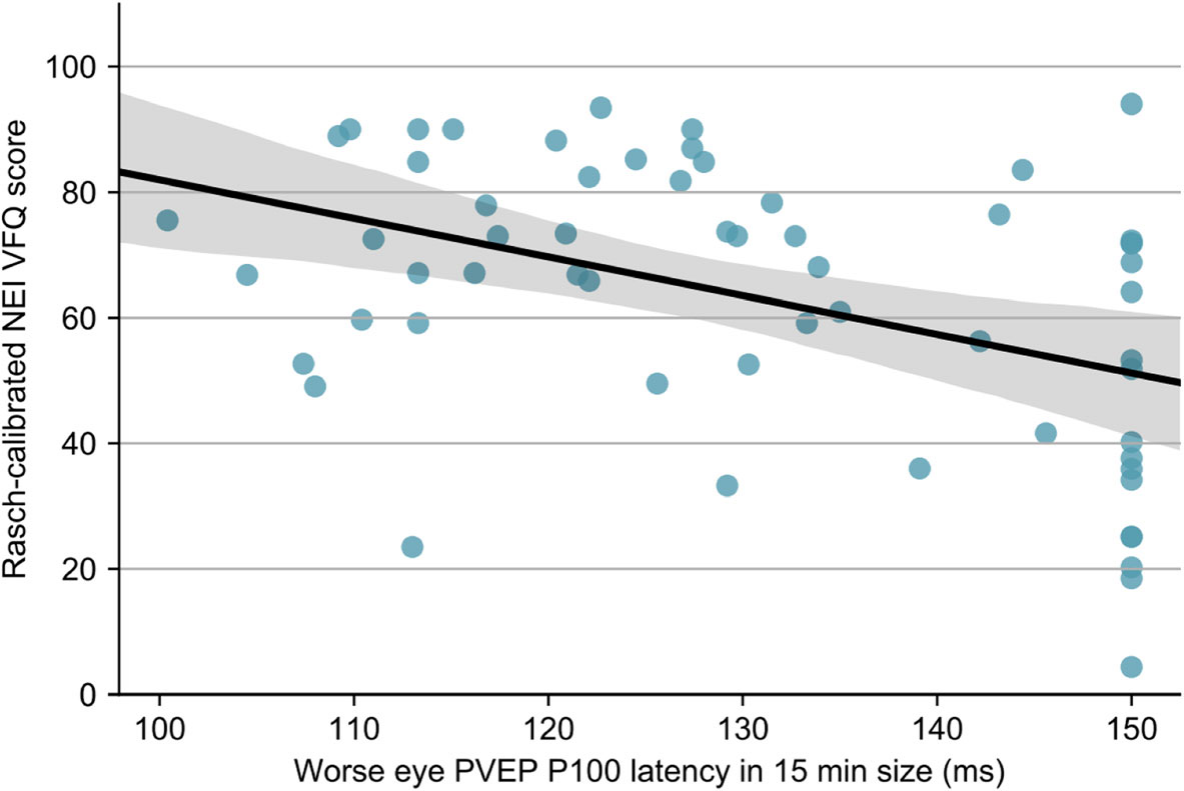
Relationship between Rasch-calbrated NEI VFQ score and PVEP P100 latency in the WE. Scatterplot of Rasch-calibrated NEI VFQ score versus PVEP P100 latency in 15 min check size in the WE. The black solid line indicates the linear regression between them (*β* = − 0.439, *P* <0.001). The gray shade represents the 95% *CI* for the regression slope

**Fig. 2:**
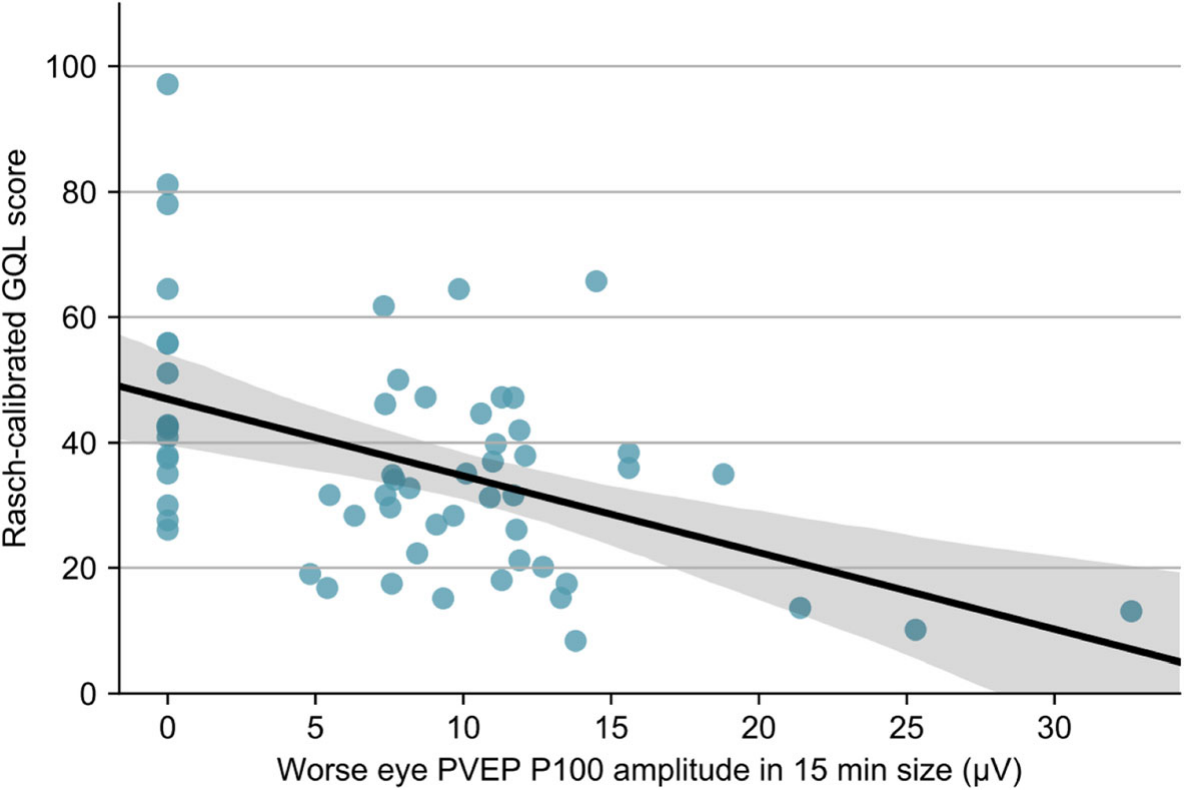
Relationship between Rasch-calibrated GQL score and PVEP P100 amplitude in the WE. Scatterplot of Rasch-calibrated GQL score versus PVEP P100 amplitude in 15 min check size in the WE. The black solid line indicates the linear regression between them (*β* = − 0.466, *P* <0.001). The gray shade represents the 95% *CI* for the regression slope

Univariate analysis revealed that worse Rasch-calibrated NEI VFQ and GQL person measure scores were associated with poorer VA, CS in both eyes, and worse VF and PVEP results in the WE (Table 2). The WE PVEP latency [*β* = − 0.439, 95% confidence interval (*CI*): (− 0.715, − 0.215), *P* < 0.001] (Fig. 1) and amplitude [*β* = 0.455, 95% *CI*: (0.223, 0.721), *P* < 0.001] in 15 min check size were moderately associated with Rasch-calibrated NEI VFQ person measure scores; The WE PVEP latency [*β* = 0.317, 95% *CI*: (0.07, 0.582), *P* = 0.014] and amplitude [*β* = − 0.466, 95% *CI*: (− 0.718, − 0.24), *P* < 0.001] (Fig. 2) in 15 min check size also had moderate associations with Rasch-calibrated GQL person measure scores. The WE MD and the WE PSD were mildly correlated with Rasch-calibrated NEI VFQ [*β* = 0.350, 95% *CI*: (0.169, 0.532), *P* < 0.001 for MD; *β* = − 0.245, 95% *CI*: (− 0.433, − 0.057)), *P* = 0.011 for PSD]and GQL [*β* = − 0.368, 95% *CI*: (− 0.548, − 0.188), *P* < 0.001 for MD; *β* = 0.204, 95% *CI*: (0.014, 0.393), *P* = 0.035 for PSD]. As for VA and CS, results in the WE had weaker associations with Rasch-calibrated NEI VFQ and GQL person measure scores compared with the BE.

**Table 2:** Univariate analysis of clinical variables and Rasch-calibrated QoL

Variables	Rasch-calibrated NEI VFQ		Rasch-calibrated GQL	
	*β* (95% CI)	*P*	*β* (95% CI)	*P*
BE logMAR VA	−0.496(− 0.664 to − 0.328)	**< 0.001**	0.575 (0.416 to 0.733)	**< 0.001**
WE logMAR VA	− 0.269(− 0.456 to − 0.083)	**0.005**	0.220 (0.031 to 0.409)	**0.023**
BE CS	0.437 (0.263 to 0.611)	**< 0.001**	−0.459(− 0.631 to − 0.287)	**< 0.001**
WE CS	0.28 (0.094 to 0.466)	**0.003**	−0.227(− 0.415 to − 0.038)	**0.019**
BE MD in VF (dB)	0.084(− 0.109 to 0.277)	0.391	− 0.135(− 0.326 to 0.057)	0.167
WE MD in VF (dB)	0.350 (0.169 to 0.532)	**< 0.001**	− 0.368(− 0.548 to − 0.188)	**< 0.001**
BE PSD in VF (dB)	−0.089(− 0.282 to 0.104)	0.361	0.113(− 0.080 to 0.305)	0.248
WE PSD in VF (dB)	−0.245(− 0.433 to − 0.057)	**0.011**	0.204 (0.014 to 0.393)	**0.035**
BE PVEP L in 1 deg. size (ms)^*^	0.066(− 0.208 to 0.348)	0.618	0.005(− 0.265 to 0.275)	0.970
BE PVEP A in 1 deg. size (μV)^*^	0.026(−0.251 to 0.306)	0.845	−0.080(− 0.351 to 0.187)	0.546
BE PVEP L in 15 min size (ms)^*^	0.013(− 0.265 to 0.292)	0.923	− 0.037(− 0.308 to 0.231)	0.776
BE PVEP A in 15 min size (μV)^*^	0.046(− 0.229 to 0.327)	0.724	−0.103(− 0.374 to 0.163)	0.434
WE PVEP L in 1 deg. size (ms)^*^	− 0.266(− 0.550 to − 0.013)	**0.040**	0.146(− 0.117 to 0.418)	0.265
WE PVEP A in 1 deg. size (μV)^*^	0.244(− 0.012 to 0.528)	0.061	−0.253(− 0.522 to 0.001)	0.051
WE PVEP L in 15 min size (ms)^*^	− 0.439(− 0.715 to − 0.215)	**< 0.001**	0.317 (0.07 to 0.582)	**0.014**
WE PVEP A in 15 min size (μV)^*^	0.455 (0.223 to 0.721)	**< 0.001**	−0.466(− 0.718 to − 0.24)	**< 0.001**

Bold items indicate *P* < 0.05 based on *t* test. *β* (95% *CI*) statistics were calculated with normalized data. ^*^: *N* = 60 for PVEP. *QoL* quality of life, *NEI VFQ* National Eye Institute Visual Function Questionnaire, *GQL* Glaucoma Quality of Life, *BE* better eye, *WE* worse eye, *MD* mean deviation, *PSD* pattern standard deviation, *VF* visual field, *PVEP* pattern visual evoked potentials, *L* latency, *A* amplitude

Stepwise multivariable linear regression was performed for all variables found to be significantly associated in the univariate model. The results revealed that prolonged WE PVEP latency in 15 min check size [*β* = − 0.345, 95% *CI*: (− 0.607, − 0.124)] and worse WE MD [*β* = 0.36, 95% *CI*: (0.134, 0.595), P] were linearly associated with decreasing Rasch-calibrated NEI VFQ person measure scores (*F* = 12.999, *P* < 0.001); lower WE PVEP amplitude in 15 min check size [*β* = − 0.343, 95% *CI*: (− 0.593, − 0.112)] and worse WE MD [*β* = − 0.345, 95% *CI*: (− 0.569, − 0.109)] were linearly associated with worse Rasch-calibrated GQL person measure scores (*F* = 13.507, *P* < 0.001) (Table 3). After adjusting for age, gender, duration of glaucoma, glaucoma severity and glaucoma type, Rasch-calibrated NEI VFQ person measure scores were only significantly associated with WE PVEP latency in 15 min check size [*β* = − 0.347, 95% *CI*: (− 0.618, − 0.118), *F* = 4.303, *P* = 0.001]; similarly, worse Rasch-calibrated GQL person measure scores were only significantly associated with WE PVEP amplitude in 15 min check size [*β* = − 0.338, 95% *CI*: (− 0.588, − 0.108), *F* = 5.169, *P* < 0.001].

**Table 3:** Stepwise multivariable linear analysis of clinical variables and Rasch-calibrated QoL (N = 60)

Variables	Rasch-calibrated NEI VFQ			Rasch-calibrated GQL		
	*β* (95% CI)	F	*P*	*β* (95% CI)	F	*P*
**Unadjusted**		12.999	**< 0.001**		13.507	**< 0.001**
WE MD in VF (dB)	0.36(0.134 to 0.595)			−0.345 (− 0.569 to − 0.109)		
WE PVEP L in 15 min size (ms)	− 0.345 (− 0.607 to − 0.124)			- -		
WE PVEP A in 15 min size (μV)	- -			− 0.343 (− 0.593 to − 0.112)		
**Adjusted** ^*^		4.303	**0.001**		5.169	**< 0.001**
WE MD in VF (dB)	- -			- -		
WE PVEP L in 15 min size (ms)	−0.347 (− 0.618 to − 0.118)			- -		
WE PVEP A in 15 min size (μV)	- -			− 0.338 (− 0.588 to − 0.108)		

Bold items indicate *P* < 0.05 based on *t* test. *β* (95% *CI*) and *F* statistics were calculated with normalized data. ^*^: Adjusted for age, gender, duration of glaucoma, glaucoma severity and glaucoma type. *QoL* quality of life, *NEI VFQ* National Eye Institute Visual Function Questionnaire, *GQL* Glaucoma Quality of Life, *BE* better eye, *WE* worse eye, *MD* mean deviation, *VF* visual field, *PVEP* pattern visual evoked potentials, *L* latency, *A* amplitude

## Discussion

In this study, we have studied the relationships between both subjective and objective clinical outcomes of visual functions and QoL in patients with glaucoma in China. We showed that on the whole the measurements of VA, CS, VF and clinical visual electrophysiology were significantly correlated with QoL in patients with glaucoma. Importantly, the QoL of glaucoma patients is multifactorial and eye dependent, as we have shown in the results, different variables of visual functions contribute differently to the QoL, therefore, it may not be appropriate to predict a patient’s QoL only through taking one or two clinical tests or just considering the better eye or the worse eye, which in some circumstances will probably lead to overlook some aspects of a patient’s QoL that are just important to him/her. Therefore, QoL measurements can be an assistant tool for clinicians in clinical decision making.

Many studies have evaluated the relationships between the outcomes of clinical examinations and the QoL in the patients with glaucoma, however, to our knowledge, most of them only focused on VF [16, 19, 21, 22, 29, 30] and / or VA [23]. In our study, we have also showed that in consistent with the previous studies, VF and VA were two associated factors affecting patients’ QoL. Interestingly, different studies concluded differently on the contributions of VF and / or VA in the BE and the WE to the QoL. Some studies found that VF and / or VA in the WE played the dominant role in the QoL [29, 31]. Some others concluded that VF and / or VA in the both eyes were good indicators for the QoL [32, 33]. However, other studies showed that VF and / or VA in the BE are the main factors related to the QoL [16, 20, 21, 30, 34]. Our data showed that the measurements of VF in the WE were significantly correlated with the QoL, in which the MD values in the WE showed stronger association with Rasch-calibrated NEI VFQ and GQL scores than the PSD values. This may be due to the fact that PSD increases with glaucoma progression during from the early stage to the middle stage, but turns into a decline after the middle stage. Compared with PSD, MD has been widely used as a parameter to monitor the glaucoma progression [35]. Therefore, it should be noted that for patients in the late stage, the use of PSD should be cautious. Meanwhile, VA in both eyes was significantly correlated with the QoL. The disparity between the results of our study and the previous studies may be attributed, at least partially, to the extent of the impairment of peripheral visual field in the WE. Our data not only showed that VF in the WE was significantly correlated with the Rasch-calibrated NEI VFQ and GQL-15 scores, respectively, but also showed that VA in the WE had weaker associations with Rasch-calibrated NEI VFQ and GQL scores compared with the BE. This is largely in line with Hirneiss’s view that when a eye disease impairs the central vision, the BE has more impact on QoL, while the peripheral vision is impaired, the clinical outcomes of the WE are better predictors [36]. The disparity between the correlations of VA and VF with QoL may be due to that the VF test is more sensitive to detect the peripheral visual deficits. However, it should be noted that overall the better eye seemed still to play an essential role in affecting the QoL in our patients since the fact that most of the daily activities need central vision.

CS is impaired in glaucoma patients [7, 8]. Recently, it was reported that the reduction of CS in patients with glaucoma correlated strongly with the changes of thickness of the retinal nerve fiber layer (RNFL) [37]. Our data showed that CS in both eyes were significantly correlated with the Rasch-calibrated NEI VFQ and GQL scores. At the same time, similar as VA, CS in the WE had weaker associations with Rasch-calibrated NEI VFQ and GQL scores compared with the BE. These results aslo imply there is a better correlation between the BE and QoL when the clinical parameter is mainly for evaluation of central visual function. Recently, a novel computer-based CS test, the Spaeth/Richman Contrast Sensitivity Test (SPARCS), which is designed to evaluate both central and peripheral vision, was invented to more reliably identify the patients with glaucoma via the internet [38]. Interestingly, Ekici et al. reported that the SPARCS had stronger correlations with the BE subscores [9]. These results suggest that the central CS take the major role in the QoL. On the other hand, it is worth mentioning that the reduction of CS in the WE may cause abnormal binocular interaction due to imbalance of visual input from the two eyes which will seriously interfere with patient’s daily living, just as the state of monocular amblyopia.

As far as we know, no study has assessed the association of an objective visual functional parameter with the QoL in patients with glaucoma. More it is worth mentioning that no study has assessed the associations of both subjective clinical outcomes and objective clinical outcomes of visual functions with QoL in a same group of glaucoma patients. In this study, we showed that both Rasch-calibrated NEI VFQ and GQL scores were significantly associated with PVEP P100 latency and amplitude in 15 min check size in the WE, but not in the BE. These results indicate on the one hand that PVEP test is a reliable examination for functional evaluation of the optic nerve damage in the WE of glaucoma patients, and on the other hand that the impairment of fine visual discrimination or the damage to the perceptual ability of high spatial resolution in the WE of glaucoma patients has a more influential impact on the patients’ QoL. More interestingly, multivariable linear regression analysis revealed that, Rasch-calibrated NEI VFQ scores were only significantly associated with PVEP latency in 15 min check size in the WE, while Rasch-calibrated GQL scores were only significantly associated with PVEP amplitude in 15 min check size in the WE. This discrepancy may reflect not only the different impact of the speed and the intensity of visual signal transmission on different dimensions of QoL, but also the difference of the scale structures of the two QoL instruments. Therefore, a combination use of these two instruments may be more appropriate for assessing the QoL of glaucoma patients and the disease progression. Overall, these results suggested that the electrophysiological measurements of visual signal transmission could be a unique method in predicting patient’s QoL.

Finally, there were some limitations in this study. Although, this is the first study to systematically examine the associations of both subjective and objective clinical outcomes of visual functions with QoL in a same group of glaucoma patients, some other clinical parameters of visual functions such as stereopsis or depth perception, colour vision and dark adaptation, were not included in the observation of this study. Correspondingly, we have not found significant correlations between the observed clinical parameters and the subscale of colour vision. At the same time, this study only focused on the visual functional deficits of glaucoma. Structural parameters such as RNFL thickness with optical coherence tomography (OCT) examination were also not included in the observation of this study.

## Conclusions

Self-reported measures of QoL could be supplemental tools for assessing both subjective and objective visual functional impairments in glaucoma patients. The profiles of correlations between different clinical parameters with QoL in glaucoma patients are different. For VA and CS, the better eye has more impact on the QoL in patients with glaucoma. For VF and PVEP, the worse eye has more influential effect on patients’ QoL. PVEP may provide unique and objective information for prediction of QoL.

## Additional file

Additional file 1: Supplementary data for Rasch analysis and demographics and clinical characteristics of all the participants. (DOCX 40 kb)

## Data Availability

The datasets used and/or analysed during the current study are available from the corresponding author on reasonable request.

## Abbreviations

AULCSF: The area under the log CS function
BE: Better eye
CACG: Chronic angle-closure glaucoma
CI: Confidence interval
CS: Contrast sensitivity
ETDRS: Early Treatment Diabetic Retinopathy Study
GQL-15: Glaucoma Quality of Life-15
ISCEV: International Society for Clinical Electrophysiology of Vision
LOCS III: The Lens Opacities Classification System III
logits: log of odds units
logMAR: logarithm of the minimum angle of resolution
MD: Mean deviation
NEI VFQ-25: National Eye Institute Visual Function Questionnaire 25
OCT: Optical coherence tomography
POAG: Primary open-angle glaucoma
PSD: Pattern standard deviation
PVEP: Pattern visual evoked potential
QoL: Quality of life
RNFL: Retinal nerve fiber layer
SITA: Swedish Interactive Threshold Algorithm
SPSS: The Statistical Package for the Social Sciences
VA: Visual acuity
VEP: Visual evoked potential
VF: Visual field
WE: Worse eye
