# Supplementary material for: Associations of subjective and objective clinical outcomes of visual functions with quality of life in Chinese glaucoma patients: a cross-sectional study
Source: BMC Ophthalmol. 2019 Jul 31;19:166. doi: 10.1186/s12886-019-1176-0 (PMC6669977; doi:10.1186/s12886-019-1176-0)
Supplement: Supplementary file 1 — Supplementary data for Rasch analysis and demographics and clinical characteristics of all the participants. (DOCX 40 kb) [file 12886_2019_1176_MOESM1_ESM.docx]

**Supplementary**

**Associations of subjective and objective clinical outcomes of visual functions with quality of life in Chinese glaucoma patients:** **a cross-sectional study**

Li Yang MD^1,2,3,4,*^, Xuefeng Shi MD, PhD^1,2,3,4,* ,†^, and Xin Tang MD, PhD^1,2,3,4^

^1^. Clinical College of Ophthalmology, Tianjin Medical University, Tianjin 300020, China.

^2^. Tianjin Eye Hospital, Tianjin 300020, China.

^3^. Tianjin Key Laboratory of Ophthalmology and Visual Science, Tianjin 300020, China.

^4^. Tianjin Eye Institute, Tianjin 300020, China.

* These authors contributed equally to this work.

† Correspondence should be addressed to Xuefeng Shi (shixf_tmu@163.com)

**eTable 1. Item content of the National Eye Institute Visual Function Questionnaire-25 questionnaire**

| **Scales** | **Item Num.** | **Items** | **Response Categories^*^** |
| --- | --- | --- | --- |
| General health | N1 | Overall health | 1=Very satisfied; 2 = Somewhat satisfied; 3 = Moderate; 4 = Somewhat dissatisfied; 5 = Very dissatisfied |
| General vision | N2 | Overall vision |  |
| Ocular pain | N3 | Having pain or discomfort in and around eyes( e.g. burning, itching or aching) | 1=Not at all; 2 = A little; 3 = Moderately; 4 = Severely; 5 = Extremely |
|  | N4 | Frequency of eye pain | 1= Never; 2 = Occasionally; 3 = Moderately; 4 = Very often; 5 = Almost always |
| Near vision | N5 | Having difficulty in reading ordinary print | 1=Not at all; 2 = A little; 3 = Moderately; 4 = Severely; 5 = Extremely |
|  | N6 | Having difficulty in doing work or hobbies such as cooking, sewing, fixing things around the house, or using hand tools |  |
| Near vision | N7 | Having difficulty in finding something on a crowed shelf |  |
| Far vision | N8 | Having difficulty in reading street signs or the name of shops |  |
|  | N9 | Having difficulty in going down steps, stairs, or curbs in dim light or at night |  |
|  | N10 | Having difficulty in going out to see movies, playing sports or other evens |  |
| Social function | N11 | Having difficulty in seeing how people react to things you say |  |
|  | N12 | Having difficulty in investing people in their homes, at parties, or in restaurants |  |
| Mental health | N13 | Thinking about the possibility of going blind |  |
|  | N14 | Concerned about doing embarrassing things because of my eyesight |  |
|  | N15 | Feeling frustrated a lot of time because of my eyesight |  |
|  | N16 | Having much less control over what I do because of my eyesight |  |
| Role function | N17 | Accomplish less than you would like to because of eyesight |  |
|  | N18 | Feeling limited in length of activities because of vision |  |
| Dependency | N19 | I stay home most of the time because of my eyesight |  |
|  | N20 | Relying too much on what other people tell me |  |
|  | N21 | Needing a lot of help from others because of my eyesight |  |
| Driving | N22 | Having difficulty in driving or riding a bike during the day |  |
|  | N23 | Having difficulty in driving or riding a bike during the night |  |
| Peripheral vision | N24 | Having difficulty in noticing objects on the side while walking straight ahead |  |
| Color vision | N25 | Having difficulty in picking out and matching clothes |  |

*: An additional category (Do not perform for nonvisual reasons) was scored as missing data for the final analysis (=0)

**eTable 2. Infit and outfit errors of NEI VFQ/ Scale structure of Rasch-calibrated NEI VFQ**

| **Item** | **General vision and social function** | |  | **Far vision** | |  | **Outdoor** | |  | **Reading and working** | |
| --- | --- | --- | --- | --- | --- | --- | --- | --- | --- | --- | --- |
|  | **Infit mean-square** | **Outfit mean-square** |  | **Infit mean-square** | **Outfit mean-square** |  | **Infit mean-square** | **Outfit mean-square** |  | **Infit mean-square** | **Outfit mean-square** |
|  |  |  |  |  |  |  |  |  |  |  |  |
|  |  |  |  |  |  |  |  |  |  |  |  |
| N2 | 0.7 | 0.72 |  | — | — |  | — | — |  | — | — |
| N5 | — | — |  | — | — |  | — | — |  | 0.57 | 0.57 |
| N8 | — | — |  | 1.15 | 1.13 |  | — | — |  | — | — |
| N9 | — | — |  | 0.53 | 0.52 |  | — | — |  | — | — |
| N10 | — | — |  | 0.69 | 0.69 |  | — | — |  | — | — |
| N11 | 1.23 | 1.16 |  | — | — |  | — | — |  | — | — |
| N12 | 0.81 | 0.77 |  | — | — |  | — | — |  | — | — |
| N17 | — | — |  | — | — |  | — | — |  | 0.8 | 0.79 |
| N18 | — | — |  | — | — |  | — | — |  | 0.96 | 0.91 |
| N19 | — | — |  | — | — |  | 1.43 | 1.3 |  | — | — |
| N22 | — | — |  | — | — |  | 0.84 | 0.79 |  | — | — |
| N23 | — | — |  | — | — |  | 1.25 | 1.26 |  | — | — |

NEI VFQ: National Eye Institute Visual Function Questionnaire.

**eTable 3. Infit and outfit errors of GQL/ Scale structure of Rasch-calibrated GQL**

| **Item** | **Detail identification** | |  | **Walking** | |  | **Adjusting to lights** | |  | **Difficult task** | |
| --- | --- | --- | --- | --- | --- | --- | --- | --- | --- | --- | --- |
|  | **Infit mean-square** | **Outfit mean-square** |  | **Infit mean-square** | **Outfit mean-square** |  | **Infit mean-square** | **Outfit mean-square** |  | **Infit mean-square** | **Outfit mean-square** |
|  |  |  |  |  |  |  |  |  |  |  |  |
|  |  |  |  |  |  |  |  |  |  |  |  |
| Reading newspapers | 1.02 | 1.02 |  | — | — |  | — | — |  | — | — |
| Walking after dark | — | — |  | 0.84 | 0.83 |  | — | — |  | — | — |
| Walking on uneven ground | — | — |  | 0.82 | 0.83 |  | — | — |  | — | — |
| Adjusting to bright lights | — | — |  | — | — |  | 1.16 | 1.17 |  | — | — |
| Adjusting to dim lights | — | — |  | — | — |  | 1.41 | 1.4 |  | — | — |
| Tripping over objects | — | — |  | — | — |  | — | — |  | 0.68 | 0.67 |
| Seeing objects coming from the side | 0.71 | 0.69 |  | — | — |  | — | — |  | — | — |
| Crossing the road | — | — |  | — | — |  | — | — |  | 0.85 | 0.83 |
| Walking on steps/stairs | — | — |  | 1.14 | 1.17 |  | — | — |  | — | — |
| Bumping into objects | — | — |  | — | — |  | — | — |  | 0.99 | 0.94 |
| Finding dropped objects | — | — |  | — | — |  | — | — |  | 0.81 | 0.76 |
| Recognizing faces | 1.36 | 1.4 |  | — | — |  | — | — |  | — | — |

GQL: Glaucoma Quality of Life.

**eTable 4. Dimensionality analysis of NEI VFQ**

|  | **Raw NEI VFQ-25 responses** | |  | **General vision and social function** | |  | **Far vision** | |  | **Outdoor** | |  | **Reading and working** | |
| --- | --- | --- | --- | --- | --- | --- | --- | --- | --- | --- | --- | --- | --- | --- |
|  | **Eigen** | **%** |  | **Eigen** | **%** |  | **Eigen** | **%** |  | **Eigen** | **%** |  | **Eigen** | **%** |
| Variance explained by measures | 19.5 | 61.90% |  | 4.3 | 59.10% |  | 13.2 | 81.40% |  | 6.3 | 67.70% |  | 22.8 | 88.40% |
| Explained by persons | 11.6 | 36.70% |  | 2.9 | 40.00% |  | 8.6 | 52.90% |  | 3.3 | 35.60% |  | 22.7 | 88.00% |
| Explained by items | 7.9 | 25.20% |  | 1.4 | 19.10% |  | 4.6 | 28.50% |  | 3 | 32.10% |  | 0.1 | 0.40% |
| Total unexplained 1st contrast | **2.5** | 7.80% |  | 1.8 | 24.20% |  | 1.9 | 12.00% |  | 2.1 | 23.00% |  | 1.4 | 5.40% |

NEI VFQ: National Eye Institute Visual Function Questionnaire; Bold item represents the unsatisfied parameter.

**eTable 5. Dimensionality analysis of GQL**

|  | **Raw GQL-15 responses** | |  | **Detail identification** | |  | **Walking** | |  | **Adjusting to lights** | |  | **Difficult tasks** | |
| --- | --- | --- | --- | --- | --- | --- | --- | --- | --- | --- | --- | --- | --- | --- |
|  | **Eigen** | **%** |  | **Eigen** | **%** |  | **Eigen** | **%** |  | **Eigen** | **%** |  | **Eigen** | **%** |
| Variance explained by measures | 14 | **53.80%** |  | 6.1 | 67.10% |  | 7.3 | 70.90% |  | 4.2 | 68.00% |  | 7.4 | 64.90% |
| Explained by persons | 8.7 | 33.50% |  | 5.3 | 58.20% |  | 5.6 | 54.70% |  | 3.3 | 52.40% |  | 6.1 | 53.60% |
| Explained by items | 5.3 | 20.20% |  | 0.8 | 8.80% |  | 1.7 | 16.20% |  | 1 | 15.60% |  | 1.3 | 11.30% |
| Total unexplained 1st contrast | 1.8 | 6.80% |  | 1.7 | 18.70% |  | 1.5 | 14.80% |  | NA | NA |  | 1.5 | 13.00% |

GQL: Glaucoma Quality of Life; NA: not available for the number of the items is not more than two.

**eTable 6. Definition of the better eye and the worse eye**

|  | **N** | **BE** | **WE** |
| --- | --- | --- | --- |
| MD in VF of the study eye is better than that of the fellow eye by ≥1dB | 91 | the study eye | the fellow eye |
| MD in VF of the study eye is better than that of the fellow eye by <1dB | 16 | the eye with better VA | the eye with worse VA |

N: number of patients; BE: better eye; WE: worse eye; MD: mean deviation; VF: visual field; VA: visual acuity.

**eTable 7. Participant Demographics and Clinical Characteristics (N = 107)**

|  | **Subjects, Mean (SD)** | | | |  |
| --- | --- | --- | --- | --- | --- |
|  | **All subjects** | **Mild** | **Moderate** | **Severe** | *P^*^* |
| Number of participants | 107 | 50 | 26 | 31 | - |
| Gender, Male (%) | 46 (42.99%) | 18 (36.00%) | 13 (50.00%) | 15 (48.38%) | 0.779 |
| Age (years) | 61.94 (10.95) | 61.78 (11.06) | 60.23 (11.97) | 63.65 (9.94) | 0.502 |
| Duration of glaucoma (years) | 3.56 (2.35) | 3.5 (2.85) | 3.96 (2.18) | 3.32 (1.445) | 0.580 |
| Type of glaucoma (%) | | | | | |
| CACG | 74 (69.16%) | 37 (74.00%) | 18 (69.23%) | 19 (61.29%) | 0.492 |
| POAG | 33 (30.84%) | 13 (26.00%) | 8 (30.77%) | 12 (38.71%) |  |
| QoL |  |  |  |  |  |
| General vision and social function | 60.18 (23.49) | 68.36 (12.72) | 58.48 (28.05) | 48.68 (27.72) | **0.001** |
| Far vision | 69.66 (28.74) | 78.85 (18.57) | 66.12 (35.53) | 57.81 (31.73) | **0.004** |
| Outdoor | 69.86 (18.02) | 75.26 (14.16) | 66.29 (24.15) | 64.12 (15.51) | **0.012** |
| Reading and working | 65.55 (23.48) | 71.34 (21.59) (n=49) | 64.72 (26.25) | 56.91 (21.87) | **0.025** |
| **NEI VFQ** | 73.47 (13.60) | 73.47 (13.6) | 63.9 (26.22) | 56.88 (21.46) | **0.001** |
| Details identification | 38.28 (22.01) | 31.83 (16.86) (n=49) | 38.01 (21.80) | 48.89 (25.81) | **0.003** |
| Walking | 38.73 (22.82) | 31 (19.72) | 38.49 (24.93) | 51.41 (20.53) | **<0.001** |
| Adjusting to lights | 36.37 (21.4) | 30.65 (19.20) | 41.88 (22.58) | 40.99 (22.08) | **0.033** |
| Difficult task | 33.17 (18.22) | 28.82 (14.18) | 33.25 (20.06) | 40.36 (20.71) | **0.022** |
| **GQL** | 30.57 (13.01) | 30.57 (13.01) | 37.91 (18.3) | 45.43 (19.38) | **0.001** |

Bold items indicate *P* < 0.05. Severity of glaucoma was classified on the basis of MD in the visual field of the worse eye as mild (-2.00 to -10.00 dB), moderate (-10.01 to -20.00 dB) and severe (< -20.00 dB). *: One-way ANOVA was used for comparison of means in the three subgroups, except for gender and glaucoma type for which chi-square test was used. SD: standard deviation; CACG: chronic angle-closure glaucoma; POAG: primary open-angle glaucoma; QoL: quality of life; NEI VFQ: National Eye Institute Visual Function Questionnaire; GQL: Glaucoma Quality of Life.
